# Longitudinal computational fluid dynamics analysis of a pediatric Kommerell diverticulum: Mechanistic insight from a case report

**DOI:** 10.1016/j.xjtc.2026.102397

**Published:** 2026-04-27

**Authors:** Yongzheng Ren, Zheyi Chu, Jianyong Huang, Yongli Cao, Chi Zhu, Nan Ding

**Affiliations:** a Department of Mechanics, School of Mechanics and Engineering Science, State Key Laboratory for Turbulence and Complex Systems, Peking University, Beijing, PR China; b Department of Radiology, Beijing Children's Hospital, Capital Medical University, National Center for Children's Health, Beijing, China; c Department of Cardiac Surgery, Beijing Children's Hospital, Capital Medical University, National Center for Children's Health, Beijing, PR China

**Figure:**
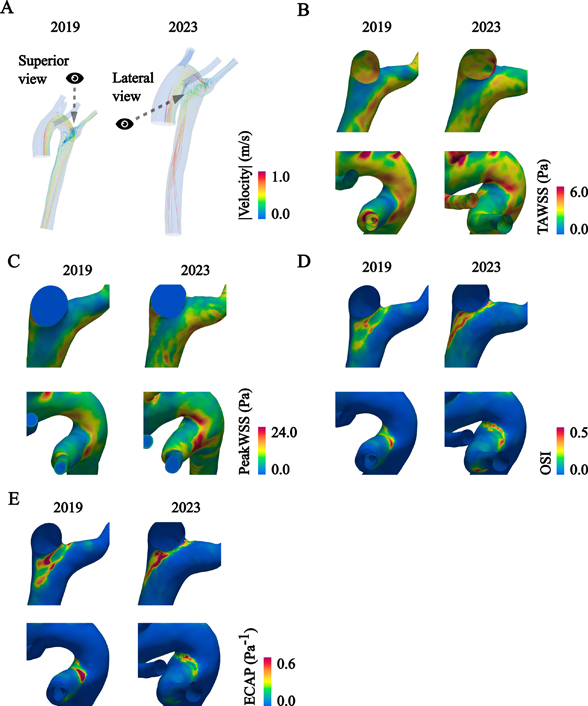
Longitudinal CFD analysis of the pediatric KD.

**Central Message**

Longitudinal CFD analysis suggests progressive adverse hemodynamics in a pediatric Kommerell diverticulum, providing mechanistic insight beyond size alone.

Kommerell diverticulum (KD) is a congenital aneurysmal dilatation at the origin of an aberrant subclavian artery and is frequently encountered in pediatric patients with right aortic arch and vascular ring. Surgical division of the ligamentum arteriosum effectively relieves airway compression in most cases. However, whether the diverticulum itself should be resected at the time of vascular ring repair remains controversial, particularly in children with modest KD size.^1^^,^^2^

Current surgical decision-making relies primarily on anatomic dimensions and symptoms, whereas tools to quantitatively assess the biological behavior of KD are limited. Computational fluid dynamics (CFD) has emerged as an investigational method to evaluate patient-specific hemodynamic environments associated with aneurysmal progression.^3^ We present a 4-year longitudinal CFD analysis of a pediatric KD to provide mechanistic insight into its hemodynamic evolution. This case illustrates how computational modeling can complement conventional anatomical evaluation by identifying progressive hemodynamic deterioration.

## Case Report

A 4-year-old boy presented in 2019 with mild exertional dyspnea. Computed tomography (CT) demonstrated a right aortic arch with an aberrant left subclavian artery arising from a KD measuring 11 × 13 mm. Airway compression was primarily attributable to the vascular ring, with possible secondary contribution from the diverticulum. Given the mild symptoms of the patient, conservative management was elected.

**Figure 1:**
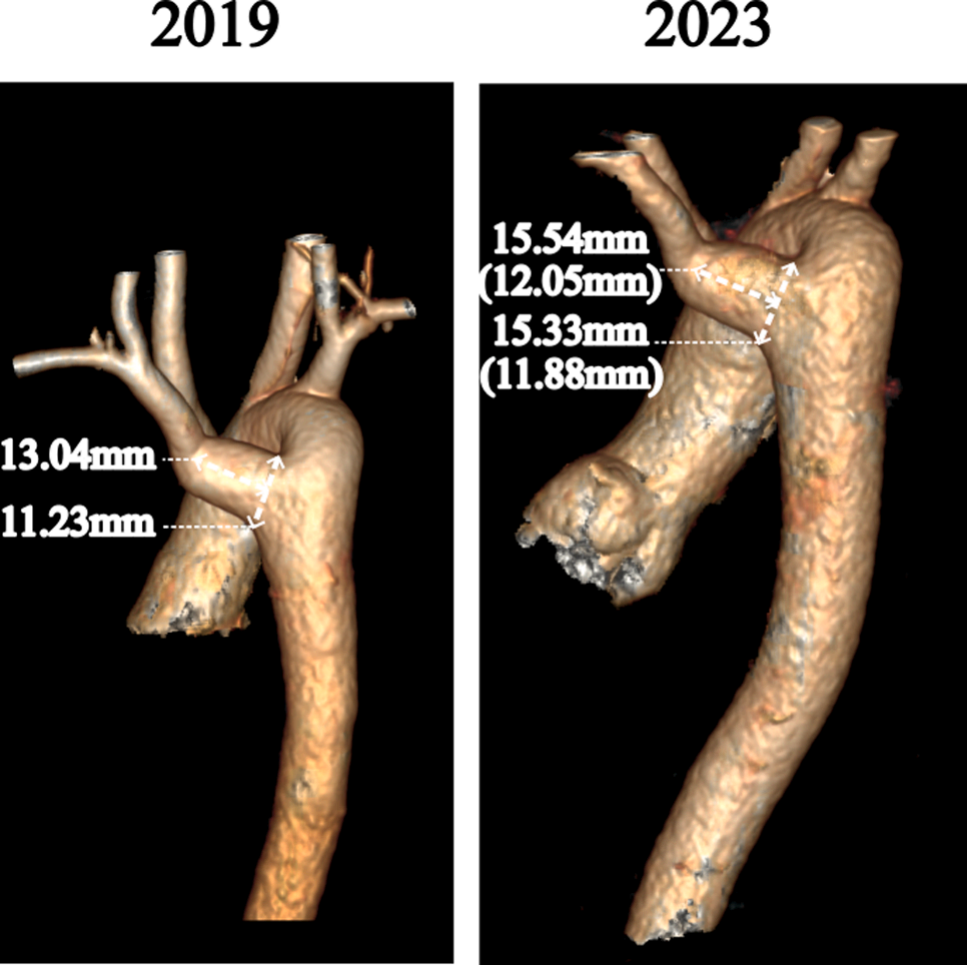
Reconstructions of patient aorta from computed tomography.

Four years later, the patient developed progressive dyspnea. To account for somatic growth, linear dimensions at follow-up were adjusted according to the square root of the ratio of body surface area between time points. Repeat CT imaging revealed enlargement of the KD to 15 × 15 mm (approximately 12 × 12 mm after adjustment for somatic growth) with persistent airway compression (Figure 1). This adjusted size was comparable with baseline dimensions, confirming that the observed growth was proportional to somatic development and that the KD did not enlarge disproportionately to overall patient growth.

**Figure 2:**
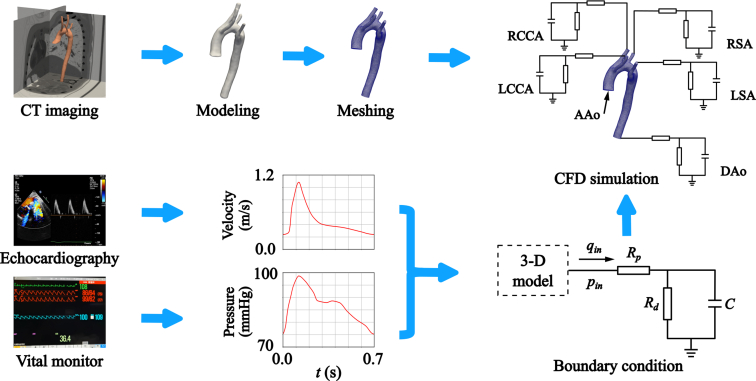
Workflow for the patient-specific simulations for the flow patterns within the diverticulum from 2019 to 2023. *CT*, Computed tomography; *RCCA*, right common carotid artery; *LCCA*, left common carotid artery; *RSA*, right subclavian artery; *LSA*, left subclavian artery; *CFD*, computational fluid dynamics; *Dao*, descending aorta; *3D*, 3-dimensional.

Patient-specific CFD simulations were performed using contrast-enhanced CT datasets from both time points. Three-dimensional vascular geometries were reconstructed using SimVascular. Hemodynamic simulations were conducted with svFSI, incorporating patient-specific inlet flow waveforms derived from echocardiography and 3-element Windkessel models applied at 5 outlets, including the descending aorta, left and right common carotid arteries, and left and right subclavian arteries. In 2019, blood pressure was 98/45 mm Hg, heart rate was 90 beats/min, and mean ascending aortic velocity was 427.5 mm/s. In 2023, blood pressure was 99/75 mm Hg, heart rate was 86 beats/min, and mean ascending aortic velocity was 454.3 mm/s. Outlet parameters were calibrated to match measured blood pressures within 5% (Figure 2).

**Figure 3:**
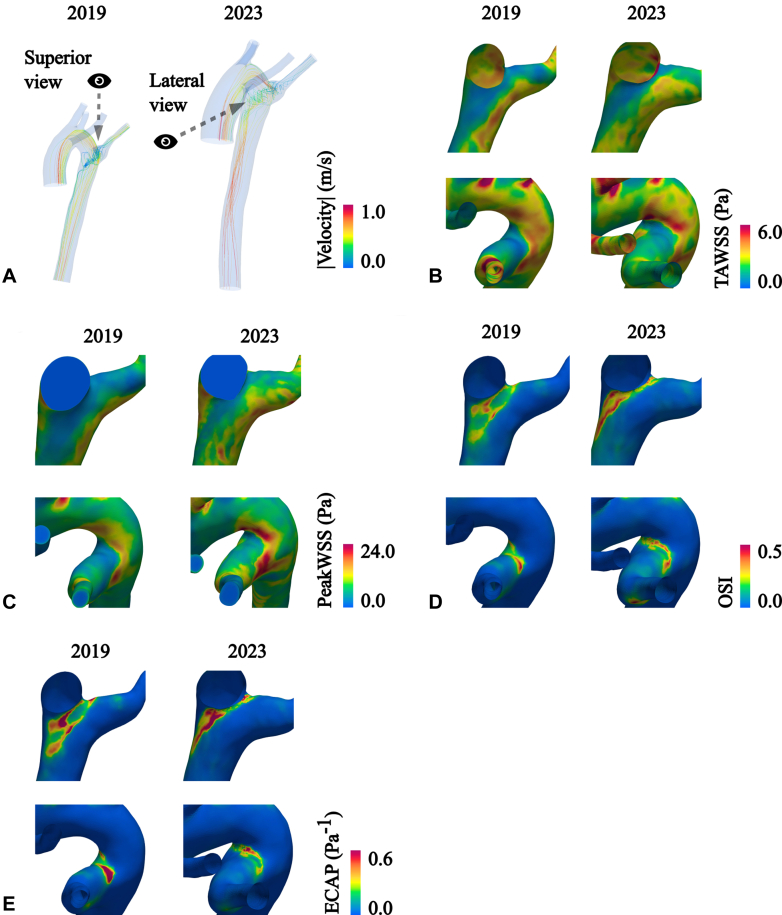
Longitudinal computational fluid dynamics analysis of the pediatric Kommerell diverticulum. A, Streamlined visualizations of vortical flow within the diverticulum. B-E, Progression of adverse hemodynamics near the diverticulum. Surface contours of $\text{TAWSS}=\frac{1}{T}\int_{0}^{T}|{\vec{\tau }}_{w}|\text{dt}$, $\text{OSI}=\frac{1}{2}\left(1-\frac{\left|\int_{0}^{T}{\vec{\tau }}_{w}\text{dt}\right|}{\int_{0}^{T}|{\vec{\tau }}_{w}|\text{dt}}\right)$ and ECAP are shown from lateral (*top row*) and superior (*bottom row*) perspectives. ${\vec{\tau }}_{w}$ is the wall shear stress, and T is the cardiac period. *TAWSS*, Time-averaged wall shear stress; *OSI*, oscillatory shear index; *ECAP*, endothelial cell activation potential.

At both time points, CFD demonstrated persistent vortical flow within the diverticulum (Figure 3, *A*). Time-averaged wall shear stress (TAWSS) revealed abnormally low stress within the KD (minimum 0.335 Pa in 2019 and 0.492 Pa in 2023) adjacent to regions of relatively high stress (maximum 5.088 Pa in 2019 and 6.866 Pa in 2023), resulting in steep spatial gradients (Figure 3, *B*). Peak wall shear stress exhibited substantial spatial heterogeneity within the KD. Regions of relatively low stress (minimum 1.596 Pa in 2019 and 2.276 Pa in 2023) were found adjacent to areas of markedly greater stress (maximum 20.506 Pa in 2019 and 24.300 Pa in 2023), creating steep spatial gradients (Figure 3, *C*).

Oscillatory shear index (OSI) peak values were similar at both time points (0.487 in 2019 and 0.488 in 2023); however, the spatial extent of elevated OSI increased over time (Figure 3, *D*). Endothelial cell activation potential (ECAP), whose formula (ECAP [Pa^−1^] = OSI/TAWSS [Pa]) integrates low TAWSS and high OSI, increased by 32% from 0.963 Pa^−1^ to 1.259 Pa^−1^, with expansion of high-risk regions within the diverticulum (Figure 3, *E*, Video 1).

**Figure 4:**
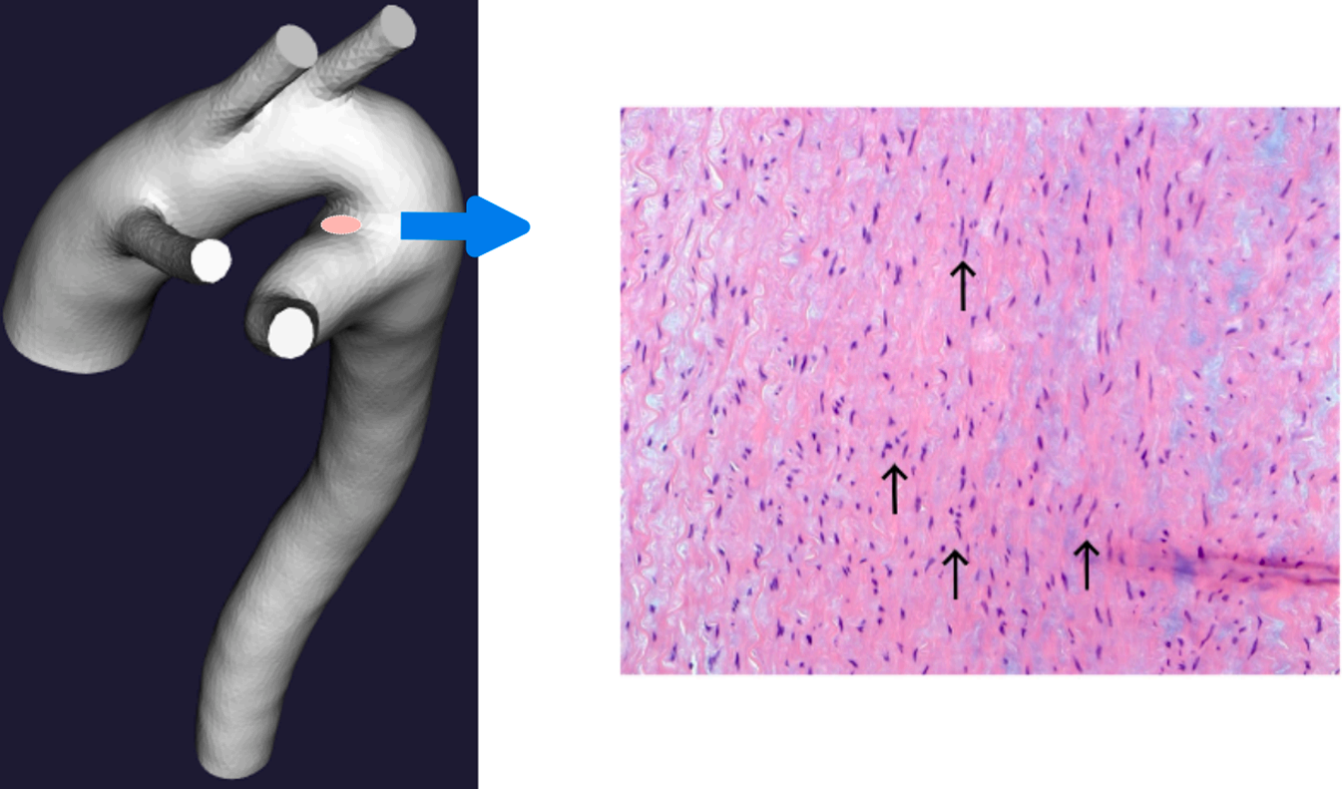
Histopathologic validation: Representative photomicrograph of the resected Kommerell diverticulum specimen (hematoxylin and eosin staining, original magnification ×40). *Black arrows* indicate focal areas of myxoid and hyaline degeneration.

The patient underwent vascular ring division with concomitant resection of the KD and reimplantation of the aberrant left subclavian artery to the left common carotid artery. Histopathologic examination of the resected KD taken from the base of the KD demonstrated focal myxoid and hyaline degeneration of the medial layer (Figure 4). The postoperative course was uneventful, and symptoms resolved completely at 18-month follow-up.

### Ethical Standards

This study was approved by Beijing Children's Hospital Ethics Committee (2023-E−041-Y, approved April 18 2023) and complies with Helsinki Declaration standards. Written informed consent was obtained from the patient's legal guardian for the publication of this study data and any accompanying images.

## Discussion

This case provides longitudinal, patient-specific hemodynamic characterization of a pediatric KD using CFD as an investigational tool. The clinical relevance lies in the integration of imaging, hemodynamics, operative findings, and histopathology within a pediatric surgical context. Notably, adverse hemodynamic features were observed despite relatively modest diverticular size.

Low TAWSS and elevated OSI have been associated with endothelial dysfunction, inflammatory signaling, and medial degeneration in various aneurysmal disease models.^4^^,^^5^ ECAP was selected in this analysis because it integrates these 2 parameters into a single, spatially intuitive metric, facilitating longitudinal comparison within the same patient. In this case, expansion of regions with elevated ECAP corresponded to histopathologic evidence of medial degeneration in the resected diverticulum.

This report does not address progression of KD after vascular ring repair and does not support routine resection of KD in all pediatric patients. Rather, it illustrates that KD may exhibit biologically adverse hemodynamics independent of airway compression, suggesting that residual diverticula should not always be assumed to be hemodynamically inert structures. Surgical decisions should continue to be individualized and guided by established clinical and anatomic criteria.

The principal limitation of this report is that it represents a single case. In addition, CFD assumptions such as rigid vessel walls may influence absolute values; however, relative longitudinal changes within the same patient are likely preserved. Further studies are required to determine whether patient-specific hemodynamic characterization can help identify subsets of KD with a propensity for progressive wall degeneration.

## Conclusions

Longitudinal CFD analysis in this pediatric case demonstrated progressive adverse hemodynamics within a KD, corroborated by histopathologic medial degeneration. These findings provide mechanistic insight into potential biological instability of KD beyond size criteria and may complement clinical decision-making in selected patients.

## Conflict of Interest Statement

The authors reported no conflicts of interest.

The *Journal* policy requires editors and reviewers to disclose conflicts of interest and to decline handling or reviewing manuscripts for which they may have a conflict of interest. The editors and reviewers of this article have no conflicts of interest.

## Supplementary Data

Video 1 Spatiotemporal distribution of WSS magnitude across the aorta during a single cardiac cycle. Video available at: https://www.jtcvs.org/article/S2666-2507(26)00204-X/fulltext.
